# Vitrectomy with internal limiting membrane peeling and gas tamponade for myopic foveoschisis

**DOI:** 10.1186/s12886-022-02376-0

**Published:** 2022-05-12

**Authors:** Jingyi Zhang, Yanping Yu, Dongshu Dai, Wu Liu

**Affiliations:** 1 Beijing Tongren Eye Center, Beijing Tongren Hospital, Capital Medical University, Beijing, 100730 China; 2 Department of Ophthalmology, Cangzhou Central Hospital, Hebei, China; 3 Beijing Ophthalmology and Visual Sciences Key Laboratory, Beijing, China

**Keywords:** Myopic foveoschisis, 23-gauge vitrectomy, ILM peeling, Gas tamponade, Foveal detachment

## Abstract

**Background:**

We evaluated the effect of vitrectomy with internal limiting membrane (ILM) peeling and gas tamponade for myopic foveoschisis (MF), and analysed prognosis with different gas tamponade.

**Methods:**

Retrospective, non-randomized study. The records of patients with MF treated by vitrectomy, were reviewed. Patients were followed up postoperatively mean 16.74 months, to record changes of Best-corrected visual acuity (BCVA) and central foveal thickness (CFT).

**Results:**

Sixty-two eyes (59 patients) were analysed in total, with mean age of 55.29 ± 10.34 years, 49 females (83.1%). Foveoschisis completely resolved in all eyes at least 6 months post vitrectomy, except for two postoperative full-thickness macular holes (FTMH). Final BCVA improved significantly from 0.69 ± 0.39 to 0.44 ± 0.42 logMAR, and CFT from 502.47 ± 164.78 to 132.67 ± 52.26 μm. Patients were subdivided into three subgroups based on the different endotamponades used (C3F8, C2F6, and air). Baseline BCVA, baseline CFT and foveal detachment (FD) were not significantly different among the three groups. Eyes treated with air tamponade had better visual outcomes than eyes with C3F8 tamponade (*P* = 0.008). Baseline BCVA and FD were significant risk factors for postoperative BCVA (*P* < 0.001 and *P* = 0.013, respectively).

**Conclusions:**

Vitrectomy with ILM peeling and gas tamponade results in good functional and anatomic outcomes in the treatment of most MF. Good vision and no-FD pre-surgery are related with good visual prognosis. Air tamponade can provide as good visual recovery as expansive gas, and reduce postoperative complications.

## Background

Myopic foveoschisis (MF) is considered one of the major causes of visual loss in highly myopic eyes [1, 2]. The estimated morbidity of MF is 8–34% in highly myopic eyes with posterior staphylomas [3, 4]. As recorded, the pathogenesis of MF is the combined effect of introversion traction and extroversion traction. Tangential vitreomacular traction is thought to be a crucial point [1, 2, 4]. Macular retinal detachment and macular hole are likely secondary complications occurring spontaneously following the development of MF, with disrupted ellipsoid zone (EZ).

The surgical options for MF include pars plana vitrectomy (PPV) and ILM peeling with or without gas tamponade, posterior scleral reinforcement alone or with vitrectomy. Moreover, the fovea-sparing ILM peeling technique has been reported to prevent iatrogenic macular holes and result in better anatomical and functional improvement than total ILM peeling [5–7]. Surgical approach, follow-up time, and the operator’s surgical skills can affect the surgical result. However, the necessity of gas tamponade or ILM peeling remains controversial [8–22].

In this retrospective study, we evaluated BCVA and CFT changes of PPV with ILM peeling and gas tamponade for MF, and compared surgical outcomes and complications between eyes with different gas tamponade.

## Methods

### Patients

A retrospective review of 62 eyes of 59 patients who underwent PPV and ILM peeling with gas tamponade for MF was performed at Beijing Tongren Hospital, between March 2012 and August 2018. The inclusion criteria were axial length > 26.0 mm, the presence of neuroretina splitting detected on a spectral-domain optical coherence tomography (SD-OCT), progressive visual symptoms (visual loss or metamorphopsia) caused by MF, follow-up ≥6 months. The exclusion criteria were other pre-existing macular pathologies, a history of ocular surgery other than cataract surgery, low quality OCT scans with insufficient resolution. Diagnosis of MF is confirmed by neuroretina splitting into a thicker inner layer and a thinner outer layer in the macula and the presence of intraretinal columns, which can be definitely detected on a SD-OCT [4]. This research followed the Tenets of the Declaration of Helsinki and was approved by the institutional review board. Data was collected from the hospital records and no patient involvement was required.

### Examinations

BCVA was measured by the tumbling E charts and converted to logarithm of the minimum angle of resolution (LogMAR) for statistical analysis. The detailed fundus was examined by indirect binocular ophthalmoscopy. Axial length was measured by IOL-Master. The macular architecture was evaluated using SD-OCT (Cirrus; Carl Zeiss, Dublin, CA). OCT images were accomplished using the ‘Macular Cube 512 × 128’ scan. The CFT was defined as the largest vertical distance measured manually between the retinal pigment epithelium and the inner retinal surface at the foveal area on horizontal OCT scan, with or without FD or lamellar hole. Other SD-OCT features were recorded: macular schisis, foveal detachment, epiretinal membranes, vitreomacular traction, lamellar holes. BCVA and OCT were recorded on all visits.

### Surgical techniques

PPV was accomplished using a 23G vitrectomy system (Alcon Constellation Vision System). Combined phacoemulsification with simultaneous intraocular lens implantation was performed on phakic eyes in case of significant cataract. The posterior cortical vitreous was removed gently from the retina after core vitrectomy. After peeling the epiretinal membrane, the ILM was peeled in all cases and 0.2 ml of 0.25% ICG staining was performed, if necessary. The ILM was entirely peeled between the upper and lower vascular arcades. Before June 2017, gas tamponade (18% C2F6 or 14% C3F8) was injected after fluid-air exchange; since June 2017, sterile air was retained in the vitreous cavity. All patients were instructed to maintain prone postoperatively, long-acting gas for 1–2 weeks, air for 5–7 days.

### Statistical methods

SPSS version 23.0 software (IBM Corp., Armonk, NY, USA) was performed for statistical analysis. Qualitative data are presented as a value, and quantitative data are expressed as the mean ± standard deviation. Independent-samples *t*-tests, Fisher’s Least Significant Difference test, Fisher’s exact test and chi-square test were used to compare the statistical significance of preoperative and postoperative outcome in BCVA, CFT, and various findings. *P* < 0.05 was considered significant.

## Results

Baseline characteristics of 62 eyes of 59 patients (M:F = 10:49) are summarized in Table 1. All patients had undergone PPV and received ILM peeling with gas tamponade. Thirty eyes (48.4%) received phacoemulsification and intraocular lens implantation simultaneously with vitrectomy. At the mean 16.74-month follow-up visit, MF completely resolved in all eyes of our study except 2 complicated FTMH. Forty-three eyes (69.4%) showed improvement in BCVA, among which 31 eyes improved two lines or more (0.3 LogMAR units). Another 12 eyes (19.4%) remained stable vision and the other 7 eyes (11.3%) suffered a vision loss, which was caused by postoperative complications (FTMH, glaucoma, and complicated cataract). The mean BCVA improved significantly from 0.69 ± 0.39 logMAR (range 0.1 to 1.5 logMAR) to 0.44 ± 0.42 logMAR (range 0.0 to 2.3 logMAR), and mean CFT improved significantly from 499.20 ± 164.38 μm (*n* = 62) to 132.67 ± 52.26 μm (*n* = 60, eyes without FTMH). (both *P* < 0.001, paired *t*-test).

**Table 1:** Baseline characteristics of the patients

Number of patients (eyes)	59 (62)
Age (years)	55.29 ± 10.34 (29 to 75)
Sex (male/female)	10/49
Duration of symptoms(months)	16.76 ± 17.25 (1 to 96)
Baseline axial length (mm)	29.31 ± 1.99 (26.23 to 34.92)
Baseline BCVA (log MAR)	0.69 ± 0.39 (0.10 to 1.52)
Baseline CFT (μm)	502.47 ± 164.78 (200 to 954)
Preoperative IOP (mmHg)	15.64 ± 3.2 (8 to 25)
Refractive error (D)	−13.09 ± 5.37 (− 1.50 to − 23.00)
Preoperative lens status (Phakic / Pseudophakic / Aphakic)	56/5/1
Epiretinal membrane	19 (30.6%)
Vitreous traction	8 (12.9%)
Foveal detachment	20 (32.3%)
Lamellar hole	9 (14.5%)
Follow-up period (months)	16.74 ± 9.40 (6 to 24)

*BCVA* best-corrected visual acuity, *log MAR* logarithm of the minimum angle of resolution, *CFT* central foveal thickness, *OCT* optical coherence tomography, *MF* myopic foveoschisis

**Fig. 1:**
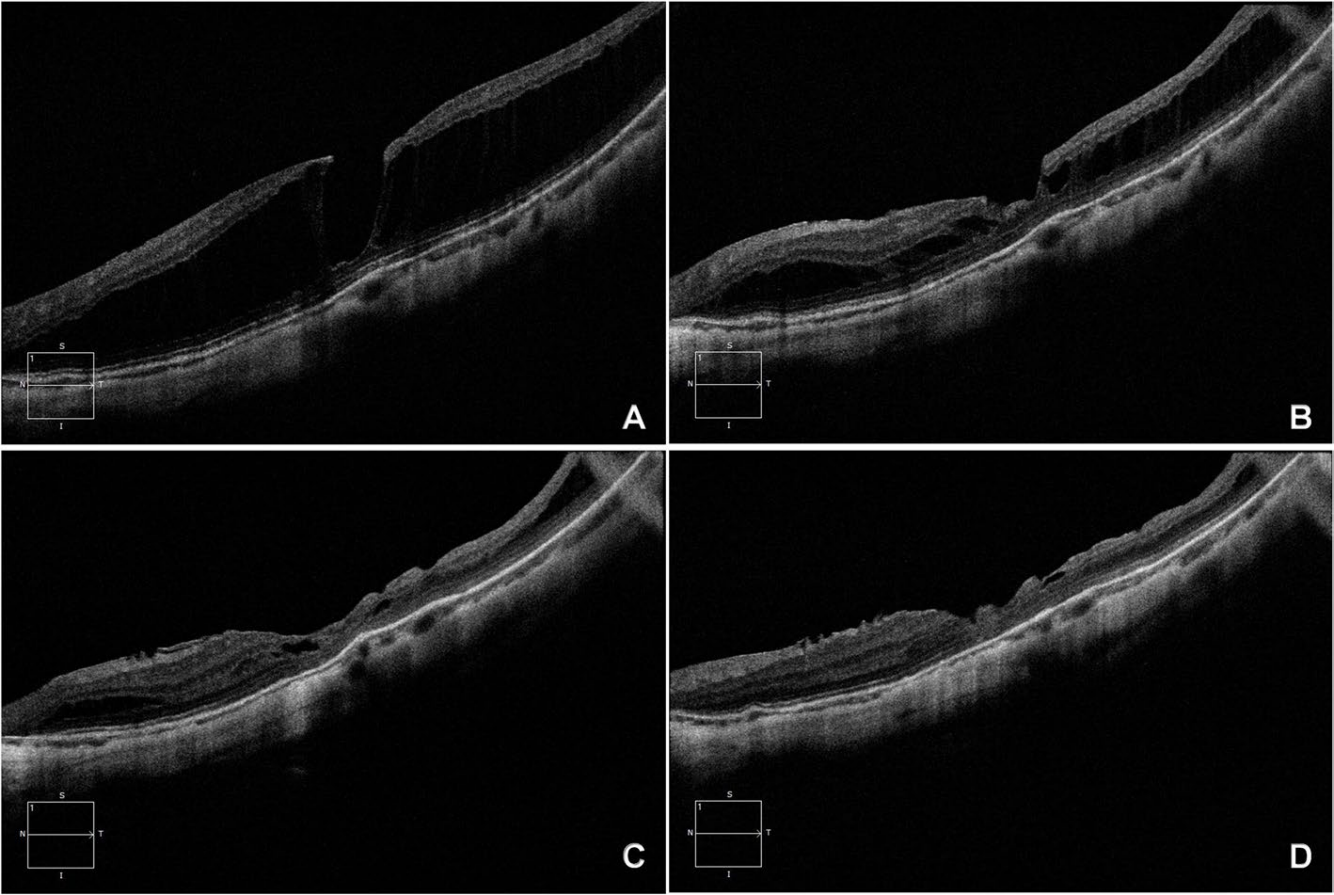
Left eye of a 63-year-old woman with an axial length of 28.89 mm, undergoing PPV with ILM peeling and C3F8 tamponade combined cataract surgery. **A** Preoperative OCT image revealed severe foveoschisis, a lamellar hole, and an epiretinal membrane, without foveal detachment . The BCVA was 0.4. **B** Postoperative OCT at 1 month showed a partial resolution with decreased area of the foveoschisis . The BCVA improved to 0.6. **C** Postoperative OCT at 3 months showed foveal resolution and residual para-foveoschisis . The BCVA was maintained at 0.6. **D** Postoperative OCT at 1 year showed a complete foveal resolution and mild temporal para-foveoschisis. The BCVA improved to 0.7

**Fig. 2:**
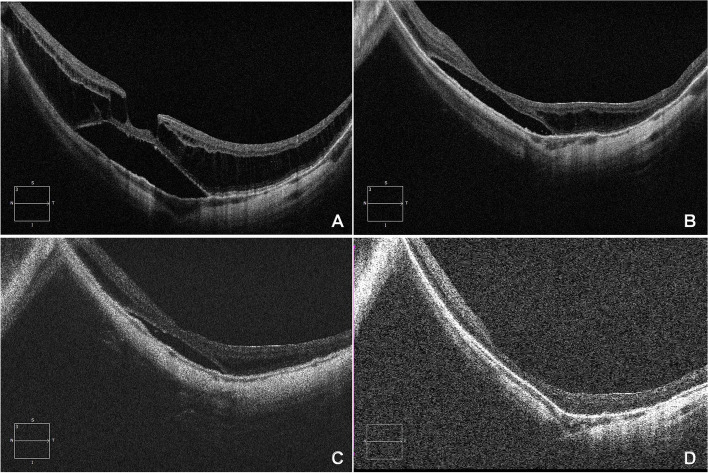
Left eye of a 54-year-old aphakic woman with an axial length of 30.7 mm, undergoing PPV with ILM peeling and air tamponade. **A** Preoperative OCT image revealed severe foveoschisis with foveal detachment. The BCVA was 0.2. **B** Postoperative OCT at 1 month showed a partial resolution with decreased height and area of the foveoshisis. The BCVA was 0.2. **C** Postoperative OCT at 3 months showed residual foveoschisis with shallow foveal detachment. The BCVA improved to 0.3. **D** Postoperative OCT at 1 year showed a complete retinal reattachment and resolution of foveoschisis. The BCVA improved to 0.4

The preoperative and postoperative parameters between MF eyes with or without FD are shown in Table 2. We originally had 42 eyes in MF without FD group, but it was necessary to delete two mild splitting cases without FD in order to reduce the effect of selection bias on the comparison of FD group and non-FD group. The eyes with FD at baseline had a thicker mean CFT (593.85 ± 178.57 vs. 461.94 ± 133.37 μm; *P* = 0.003) and a worse mean BCVA (0.91 ± 0.34 vs. 0.61 ± 0.37 logMAR; *P* = 0.005) preoperatively, compared to those without FD at baseline. Both groups had significant improvement in CFT reduction and BCVA (Figs. 1 and 2). However, eyes with FD at baseline had a thinner mean CFT (113.60 ± 55.36 vs. 144.74 ± 48.44 μm; *P* = 0.034) and a worse mean BCVA (0.60 ± 0.36 vs. 0.36 ± 0.44 logMAR; *P* = 0.043) postoperatively.

**Table 2:** Parameters before and after surgery between eyes of myopic foveoschisis with or without foveal detachment

Parameters	MF without FD (*n* = 40)	MF with FD (*n* = 20)	*P* value
Baseline CFT (μm)	461.94 ± 133.37	593.85 ± 178.57	0.003^a^
Post-op CFT (μm)	144.74 ± 48.44	113.60 ± 55.36	0.034^a^
Change CFT (μm)	304.91 ± 128.33	480.25 ± 192.18	0.000^a^
Baseline BCVA (log MAR)	0.61 ± 0.37	0.91 ± 0.34	0.005^a^
Post-op BCVA (log MAR)	0.36 ± 0.44	0.60 ± 0.36	0.043^a^
Change BCVA (log MAR)	−0.25 ± 0.47	−0.31 ± 0.29	0.622^a^
Gas tamponade (C3F8/C2F6/Air)	19/8/13	12/5/3	0.389^b^

*BCVA* best-corrected visual acuity, *log MAR* logarithm of the minimum angle of resolution, *CFT* central foveal thickness, *FD* foveal detachment

^a ^Independent-Samples t-test

^b^ χ^2^ test

Three categories grouped based on the different endotamponades used (C3F8, C2F6, and air) are shown in Table 3. We deleted 7 cases with postoperative complication in order to analyze the improved effect of 3 gases on the structural and functional aspects of MF, without the influence of extreme data (2 FTMH, 1 glaucoma, and 4 complicated cataract). Twenty-eight eyes (50.9%) used C3F8 tamponade, 11 eyes (20.0%) C2F6 tamponade, and the remaining 16 eyes (29.1%) air tamponade. There were no statistically significant differences in age, axial length, CFT, FD, baseline BCVA, change BCVA or cataract treatment in these three groups. However, postoperative BCVA was statistically different among these three groups (*P* = 0.01, Least Significant Difference test). Eyes treated with air tamponade had better visual outcome than eyes with C3F8 tamponade, with *P* value of 0.008 (Least Significant Difference test). Postoperative BCVA was not statistically different between groups treated with C2F6 and C3F8 tamponade, nor between C2F6 and air tamponade (*P* = 0.989, *P* = 0.019, respectively, Least Significant Difference test). The follow-up period wasn’t statistically different between C3F8 and air tamponade (*P* = 0.655), but it was significantly longer in C2F6 group than in the other two groups (*P* = 0.013, *P* = 0.010, respectively, Least Significant Difference test).

**Table 3:** Characteristics of patients treated with different gas tamponades

Parameters	C_3_F_8_ (*n* = 28)	C_2_F_6_ (*n* = 11)	Air (*n* = 16)	*P* value
Age (years)	53.96 ± 10.43	59.09 ± 9.39	56.88 ± 9.84	0.327^a^
Sex (M/F, eyes)	8/20	0/11	2/14	
Axial length (mm)	29.41 ± 2.36	29.30 ± 1.23	28.86 ± 1.36	0.657^a^
Follow-up period (months)	15.86 ± 8.20	23.82 ± 12.02	14.63 ± 6.92	0.021^a^
Baseline CFT (μm)	520.93 ± 170.79	477.91 ± 198.68	487.00 ± 147.45	0.731^a^
Post-op CFT (μm)	126.18 ± 50.23	124.64 ± 47.96	162.25 ± 57.80	0.111^a^
Change CFT (μm)	396.11 ± 182.52	353.27 ± 191.86	324.75 ± 179.74	0.511^a^
Baseline BCVA (log MAR)	0.82 ± 0.39	0.61 ± 0.25	0.61 ± 0.42	0.142^a^
Post-op BCVA (log MAR)	0.54 ± 0.37	0.25 ± 0.24	0.25 ± 0.33	0.010^a^
Change BCVA (log MAR)	0.28 ± 0.27	0.37 ± 0.25	0.36 ± 0.39	0.581^a^
Foveal detachment	12	5	3	0.252^b^

*CFT* Central foveal thickness, *logMAR* Logarithm of the minimum angle of resolution, *BCVA* Best corrected visual acuity. All values are shown as the mean ± standard deviation

^a^ Fisher’s Least Significant Difference test

^b^ Fisher’s exact test

Factors affecting the change in BCVA using multiple linear regression analysis are shown in Table 4. Better postoperative BCVA had a high correlation with better baseline BCVA and absence of FD (*P* < 0.001, 0.013, multiple linear regression analysis), whereas age, duration of symptoms, axial length, baseline CRT, phacoemulsification, epiretinal membrane, vitreous traction and Lamellar hole were not correlated with final visual outcome. Baseline BCVA (*β* = 0.579) had greater effect on postoperative BCVA than foveal detachment (*β* = 0.316).

**Table 4:** Correlation between preoperative factors and final postoperative BCVA

Preoperative factors	*P*	*β*
Age	0.617	0.073
Duration of symptoms(months)	0.517	−.086
Axial length	0.311	0.120
Baseline BCVA	^1^< 0.001	0.579
Baseline CFT (μm)	0.336	−.123
Phacoemulsification	0.226	−.161
Epiretinal membrane	0.644	0.057
Vitreous traction	0.608	0.065
Foveal detachment	^1^0.013	0.316
Lamellar hole	0.799	0.033

*BCVA* Best-corrected visual acuity, *CRT* Central foveal thickness.^1^Statistically significant (multiple linear regression, stepwise)

### Complications

FTMH developed in 2 eyes (3.2%). One eye with C2F6 tamponade, FTMH was present at 1 month after surgery, maintained stable postoperative vision without macular detachment or the macular hole progression in 1 year follow-up. Another eye with C3F8 tamponade, FTMH was present at 4 month after surgery, complicated with retinal detachment in the 11th month after surgery, underwent reoperation with silicone oil tamponade. The retina was reattached and MH was closed in the second FTMH eye. One eye developed glaucoma with baseline intraocular pressure (IOP) of 23 mmHg, axial length of 34.92 mm and with C3F8 tamponade (1.6%), recovered with anti-glaucoma medication. Of the 32 eyes in which the lens showed clear preoperatively, cataract developed in 18 eyes (56%), 14 eyes were cured by cataract removal during the follow-up period. The remaining four eyes with postoperative vision deterioration are still under observation. For these 18 eyes, 12 eyes were with C3F8 tamponade, 4 eyes with C2F6 tamponade, and 2 eyes with air tamponade.

### Lens status

At the last follow-up, a total of 44 eyes underwent cataract surgery, 5 pseudophakic eyes, 1 aphakic eye, with the remaining 12 being phakic (4 opacity vs. 8 transparent).

## Discussion

The results of our study showed that most patients with MF and progressive visual symptoms may obtain anatomical and functional improvement from 23-gauge vitrectomy with ILM peeling at least 6 months following (*p* < 0.001). Many earlier studies confirmed that PPV with ILM peeling results in better BCVA and anatomic outcomes compared with PPV alone [5, 8, 9, 13, 14, 23–25]. However, some researchers hypothesized that total ILM peeling could increase the risk of developing a FTMH, macular shift and inner retinal dimples [5, 26]. FTMH is a common complication after PPV with ILM peeling for MF, with an incidence of about 12.5% ~ 27.3%. In our study, 2 of 62 eyes (3.2%) developed FTMH with total ILM peeling, similar to the incidence of PPV without ILM peeling studies and ‘Fovea-sparing ILM peeling’ studies [27–29]. ‘Fovea-sparing ILM peeling’ method was raised by Ho [7] and Shimada [5] to prevent the formation of FTMH and to reduce the damage to the structure of macular after vitrectomy. It’s extremely challenging to preserve a small size of spared-fovea ILM with long axial length. If the size is not small enough or the margin is not sharp, the preserved ILM itself may cause a late contraction [5]. ILM peeling, gas tamponade, and preoperative ellipsoid disruption may be risk factors of the formation of FTMH [13–15, 20, 22, 30, 31]. It has been reported that postoperative FTMH is more common in MF with FD [2, 32, 33]. Two of 9 preoperative lamellar MHs in our study that developed postoperative FTMH (one case with C3F8 tamponade, the other one with C2F6 tamponade). Tian’s research showed that 1 of 2 (50%)preoperative lamellar MHs in the fovea-sparing ILM peeling group developed postoperative FTMH and 1 (100%) in the total ILM peeling group. His research believed that the only risk factor for development of postoperative FTMH was preoperative outer lamellar MH, regardless of the surgical procedure selection, fovea-sparing or total ILM peeling [29]. However, in Shimada’s [5] and Ho’s studies [34], no postoperative development of FTMH was found in the fovea-sparing ILM peeling group. We hypothesized that the foveal tissue with lamellar MH or EZ defect is fragile and is more easily damaged by the traction of the surgical retinal reattachment to posterior staphyloma. How to reduce the occurrence of postoperative MH requires further study.

Overall, 43 (69.4%) eyes showed improvement in BCVA in our study. Seven eyes had vision decrease, which was mainly caused by FTMH and complicated cataract. Cataract progression was seen in 18 eyes in our group, of which 14 eyes underwent subsequent cataract surgery and 4 eyes didn’t because of the patients’ own requirement and were under observation in the period of follow-up. The study of Rishi et al. reported that age of the patient, type of intraocular gas, duration of prone positioning, and the effective time of contact of gas bubble with the crystalline lens may be several factors influencing the cataract progression after PPV and gas tamponade [35]. We analyzed BCVA change of 14 eyes with cataract progression in the postoperative period. The majority of 14 eyes had a changing trend in having postoperative vision rising first and then decreasing with progression of cataract; meanwhile, MF had continuous structural improvement. After the subsequent cataract surgery, BCVA of 14 eyes return to the optimal level again during the follow-up period. On balance, foveoschisis resolution was the basis of good postoperative vision, cataract surgery enabled further visual recovery. For patients with preoperative transparent lens, it is necessary to inform them of the possibility of postoperative cataract progression and treat the cataract in time.

In our study, the eyes with FD had a thicker baseline mean CFT and a thinner postoperative mean CFT, compared with those without FD, probably due to EZ disruption preoperatively related to the foveoschisis and FD. This disruptive change may bring about worse preoperative BCVA and postoperative BCVA, compared with those eyes without FD at baseline [31]. Our study showed that a better baseline BCVA and absence of FD were significantly correlated with a better visual prognosis (*p* < 0.001), similar to the results by Lee et al. [36] and Al-Badawi et al. [37]. Nonetheless, even eyes with FD achieved as much improvement in vision and macular structure as eyes without FD from baseline. Therefore, when the central fovea is affected, with vision decline, it is effective to take surgical treatment of vitrectomy in time to restore the foveal configuration and prevent macular hole and retinal detachment. For eyes with FD with long-term low vision, surgery is still an effective way to improve visual acuity.

Whether or not to use gas tamponade and which gas is more suitable for tamponade has been controversial [14, 20, 38]. Jiang et al. [39] considered that C3F8 was more effective in patients than air. Hwang et al. [18] reported that there was no significant difference in postoperative BCVA between air tamponade group and C3F8 group. In this study, air, C2F6 and C3F8 tamponade were divided into three groups according to different tamponade material. There was no significant difference in FD between the three groups (*P* = 0.252), which could exclude the effect on the statistical results. All eyes of the three groups showed significant visual improvement after vitrectomy. The air tamponade group showed a greater improvement in postoperative BCVA compared with the C3F8 tamponade group. This was consistent with a better preoperative BCVA and a lower proportion of FD in air tamponade group, although there was no significant difference between the two groups. Moreover, there was no significant difference in the BCVA change between the two groups, indicating that air tamponade was as effective as C3F8 tamponade in treating MF. C2f6 provided tamponading effect for an intermediate duration in the 3 gases, which may be the reason of no significant difference in postoperative BCVA between groups treated with C2F6 and C3F8 tamponade, nor between C2F6 and air tamponade. The longest period of follow-up of C2F6 group may influence little on the comparison of postoperative BCVA among the three groups.

There are several limitations to this study. First, although our surgical technique was standardised, the decision to use either air or expansive gas intraocular tamponade depended on the surgical period (by June 2017) and the surgeon’s experience rather than being driven by particular guidelines. Second, we didn’t note EZ disruption in the study, because some OCT scans were of poor quality caused by lens opacity and extreme axial length. Third, the sample size was not enough, and the patients were not randomized into groups.

## Conclusions

Our study demonstrated that a better baseline BCVA and absence of FD were strongly correlated with a better visual prognosis in the treatment of MF. Most patients with MF may obtain anatomical and functional improvement from vitrectomy with ILM peeling and gas tamponade, regardless of whether FD is present at baseline. Air tamponade can provide as good visual recovery as expansive gas, and reduce postoperative complications.

## Data Availability

The datasets used and analysed during the current study are available from the corresponding author on reasonable request.

## Abbreviations

ILM: Internal limiting membrane
MF: Myopic foveoschisis
BCVA: Best-corrected visual acuity
CFT: Central foveal thickness
SD-OCT: Spectral-domain optical coherence tomography
MH: Macular hole
FTMH: Full-thickness macular holes
FD: Foveal detachment
EZ: Ellipsoid zone
PPV: Pars plana vitrectomy
LogMAR: Logarithm of the minimum angle of resolution
IOP: Intraocular pressure
